# Physiological Response of *Escherichia coli* W3110 and BL21 to the Aerobic Expression of *Vitreoscilla Hemoglobin*

**DOI:** 10.4014/jmb.2004.04030

**Published:** 2020-07-14

**Authors:** Alvaro R. Lara, Janet Galindo, Karim E. Jaén, Mariana Juárez, Juan-Carlos Sigala

**Affiliations:** Departamento de Procesos y Tecnología, Universidad Autónoma Metropolitana-Cuajimalpa, Vasco de Quiroga 4871, Santa Fe, CP 05348, Mexico City, Mexico

**Keywords:** *Vitreoscilla hemoglobin*, aerobic cultures, *E. coli* W3110, BL21, transcriptional analysis

## Abstract

The aerobic growth and metabolic performance of *Escherichia coli* strains BL21 and W3110 were studied when the *Vitreoscilla* hemoglobin (VHb) was constitutively expressed in the chromosome. When VHb was expressed, acetate production decreased in both strains and was nearly eliminated in BL21. Transcriptional levels of the glyoxylate shunt genes decreased in both strains when VHb was expressed. However, higher transcription of the α-ketoglutarate dehydrogenase genes were observed for W3110, while for BL21 transcription levels decreased. VHb expression reduced the transcription of the cytochrome bo_3_ genes only in BL21. These results are useful for better selecting a production host.

A well-established technology to cope with O_2_ limitation is the expression of *Vitreoscilla hemoglobin* (VHb). Possible actions of VHb are the binding to O_2_ and its delivery to the respiratory chain [1-3]. However, the effect of VHb under aerobic conditions has been only scarcely studied. The VHb-enhanced respiratory activity may increase the fluxes in the tricarboxylic acid cycle (TCA), lowering the aerobic acetate production [4]. As a result, aerobic VHb expression reduced acetate production in several *Escherichia coli* strains [4], lactate production by CHO cells [5], and the lipids production in *Yarrowia lipolytica* [6]. The impact of VHb expression in aerobic cultures of the strains *E. coli* W3110 and BL21, relevant for industrial and academic purposes [7, 8], was studied here. To avoid gene copy variations arising from plasmid-based expression, a single *vgb* gene copy (coding for VHb) was inserted in the chromosome of BL21 and W3110 and constitutively expressed. The effect of VHb expression was studied in batch cultures (Fig. 1). W3110 produced acetate concomitantly with biomass formation and glucose consumption. At the point of glucose depletion, acetate concentration, oxygen transfer (OTR) and carbon dioxide evolution (CER) rates reached their maximum (Fig. 1C). OTR and CER rapidly decrease thereafter, indicating no O_2_ limitations. Then, both rates increased transiently, coincident with acetate re-assimilation (Fig. 1A). When VHb was expressed in W3110, the profiles were similar (Figs. 1B and 1D), but around 30% less acetate was accumulated, in comparison with the non-expressing strain. In the case of strain BL21, low amounts of acetate were accumulated (Fig. 1E). Accordingly, no second peaks of OTR and CER were detected after glucose depletion (Fig. 1G). Strain BL21 is well known for its low acetate accumulation and is amply used for high cell-density processes [10]. The low acetate production by BL21 has been attributed to a more active higher expression of glyoxylate shunt genes, compared with K12-derived strains [10, 11]. When VHb was expressed in BL21, acetate accumulation was nearly eliminated (Fig. 1F). Minor amounts of lactate and succinate were found in the samples.

Despite using the same expression system, the active VHb concentration was more than three-fold higher for BL21 than for W3110 (Fig. 2), which may be attributed to a lower protease activity in BL21, compared to W3110 [7]. The amounts of active VHb found are small compared to previous reports that range from 13-238 nmol gWCW^-1^ [11] to 3.4-3.8 mmol gDCW^-1^ (*ca*. 850-950 nmol gWCW^-1^) [12] using high-copy number plasmids. Nevertheless, the comparatively small amount of VHb caused relevant physiological changes, as detailed below. While VHb expression did not affect the specific growth rate (μ) in W3110, it was *ca*. 15 % lower in BL21*vgb*^+^, compared to its parent strain (Table 1). The acetate yields decreased by 30 and 60% in W3110 and BL21, respectively, when VHb was expressed. The specific glucose uptake rate (*q_S_*) was *ca*. 12% higher in BL21 than in W3110 wild-types (Table 1). Marisch *et al*., [7] demonstrated that strain BL21 displays a higher expression of genes involved in glucose transport than K-12 strains. Namely, genes coding for the galactiol PTS permease (*gatABC*), mannose PTS permease (*manXYZ*), maltose ABC transporter (*malKFGE*), and LamB porin (*lamB*) were overexpressed in BL21, in comparison with a K12 derived strain [7]. This can contribute to the higher *q_S_* observed for BL21, compared to W3110. When VHb was expressed in W3110, *q_S_* increased by 19 %, compared to the wild-type (Table 1). This may point to an increased glucose oxidation capacity. Growth rate reduction, together with increased *q_S_* and *q_O2_* have been also reported for evolved K-12 derivatives [14]. A slight *q_S_* decrease (*ca*. 8%) was observed when VHb was expressed in strain BL21. Such a small decrease *q_S_* under aerobic conditions is in agreement with previous results expressing VHb from a high copy-number phagemid under aerobic conditions [4]. Other authors reported a 27% decrease of *q_S_* when VHb is expressed from a plasmid in strain MG1655 during microaerobic fed-batch cultures [15]. The *q_S_* decrease observed only in BL21 could be linked to the higher VHb expression, compared to W3110, and growth rate reduction. The specific rate of acetate production (*q_acet_*) decreased in the strains expressing VHb. Nonetheless, the specific rate of lactate production, which was unaffected by VHb in W3110, increased significantly when VHb was present in BL21. Lactate production under aerobic conditions has been reported for BL21 and attributed to overflow metabolism (excretion of partially oxidized carbon molecules under aerobic conditions) [7, 16]. When aerobic acetate production was diminished through inactivation of the involved pathways, lactate production increased [16], coincident with the results of Table 1. The effect of VHb on specific rate of succinate production was negligible for W3110 but in BL21 decreased by half. The specific oxygen uptake (*q_O2_*) and CO_2_ production (*q_CO2_*) rates in W3110 increased by 20%when VHb was expressed, in agreement with its hypothetic function. The estimated *q_ATP_*, directly proportional to *q_O2_*, also indicates a higher energy generation rate in W3110*vgb*^+^ and BL21*vgb*^-^, compared with W3110*vgb*^-^ and BL21*vgb*^+^, respectively.

The transcription level of *zwf* gene, coding for the first enzyme of the pentose phosphate pathway (PPP) was nearly two-fold in W3110*vgb*^+^, but was slightly lower in BL21*vgb*^+^, compared with their parent strains (Fig. 3). This may indicate a change in the flux to the PPP, since the expression of *zwf* is consistently linked to the flux in the corresponding pathway [16]. This is consistent with flux balance analysis performed by Tsai and coworkers [12], who estimated a higher flux through the PPP when VHb was expressed in strain W3110 under microaerobic conditions. However, a similar hypothesis about the flux through the PPP in BL21 cannot be formulated with the available information. Nevertheless, it is known that *zwf* expression is growth-rate regulated [17], which is in agreement with the lower μ in BL21*vgb*^+^. The acetate forming pathways were slightly affected by the presence of VHb in W3110. While the *poxB* expression was unaffected in both strains, *pta* expression in BL21 was slightly repressed. This is in agreement with the observed reduction of acetate production. Genes from the glyoxylate shunt in BL21 were also slightly repressed when VHb was present. This may be linked to a lower re-assimilation of acetate during cell growth, consequence of a lower acetate production. The genes coding for enzymes of the oxidative decarboxylation of α-ketoglutarate (*sucA* and *sucB*) were overexpressed in W3110*vgb*^+^, relative to its wild-type. *sucA* transcription level is also consistently linked to the flux in the involved reaction [18]. The reaction involved produces CO_2_, which is in agreement with the observed increase of *q_CO2_* in W3110*vgb*^+^ (Table 1). In contrast, the expression of *sucA* and *sucB* was lower in BL21*vgb*^+^, relative to its wild-type, in agreement with the *q_CO2_* decrease on (Table 1). Moreover, the decarboxylation of α-ketoglutarate produces NADH. The lower NADH synthesis expected in strain BL21 as effect of VHb expression, can be linked with a reduced transmembrane proton gradient. As consequence, less ATP is produced (Table 1). *cyoA* and *cyoB* code for components of the cytochrome bo_3_, which is the cytochrome prevailing under aerobic conditions in *E. coli*. The transcription levels of such genes were not affected by VHb in W3110. In contrast, *cyoB* was repressed in BL21. This may be related with a lower NADH regeneration NADH by SucA (1 NADH mol per succinyl-CoA mol synthetized), which is a key cofactor driving the respiratory chain and consistent with the lower *q_O2_* observed (Table 1). The results show that the effects of aerobic VHb expression will depend on the particular strain used, and that different advantages (like reduced acetate accumulation) and potential disadvantages (like lower growth rate in BL21) may arise.

## Materials and Methods

### Strains and Culture Conditions and Off-Line Analyses

The *Escherichia coli* strains used in this study were W3110*recA*^-^ and BL21(DE3) *recA*^-^. The *vgb* gene, coding for the *Vitreoscilla stercoraria* hemoglobin was synthesized by GeneScript (USA). Then, it was cloned in pLoxGen*trc* between the NcoI and EcoRI sites downstream the *trc* promoter (P_*trc*_). The DNA region comprising P_*trc*_, *vgb* and gentamicin resistance genes flanked by Lox sequences was then PCR amplified from the using the high-fidelity polymerase Phusion (ThermoScientific, USA) and oligonucleotides 5´-GTTATTTCTTGATGTCTCTGACCA GACACCCATCAACAGTATTATTTTCTAGCTTATCATCGACTGCACG-3´and 5´-GACATGGCCTGCCCG GTTATTATTATTTTTGACACCAGACCAACTGCACAGATGCGTAAGGAGAA-3´ [19, 20]. The resulting 2,487 nt product was used to transform the *E. coli* strains bearing the plasmid was pKD46, in which the Red system proteins were already induced with arabinose. This way, the PCR product was integrated into the chromosome by homologous recombination between *lacI* and *lacZ*. The integration was confirmed by resistance to gentamicin and the absence of blue coloration in colonies grown in LB agar containing X-Gal 10 μg/ml and IPTG (1 mM). PCR tests demonstrated successful integration of *vgb*. Engineered cells were inoculated in 250 ml shake-flasks containing 50 ml of mineral medium during 12-16 h, in orbital shakers at 250 rpm and 37°C. The composition of the medium is as follows: K_2_HPO_4_, 17; KH_2_PO_4_, 5.3; (NH_4_)_2_SO_4_, 2.5; NH_4_Cl, 1.0; Citrate-Na_3_·2H_2_O, 2; MgSO_4_·7H_2_O, 1.0; Thiamine-HCl, 0.01. The medium was supplemented with trace element solution (2 ml/l) and kanamycin sulfate (50 μg/ml). The trace element solution composition (in g/l) was ZnCl_2_, 10.5; EDTA, 5.5; CoSO_4_·7H_2_O, 1.5; MnSO_4_·H_2_O, 6.4; CuSO_4_·5H_2_O, 1.1; H_3_BO_3_, 1.5; Na_2_MoO_4_·2H_2_O, 1; FeCl_3_·6H_2_O, 51.4; and Cit-H·H_2_O, 39.9. The broth was collected and used to inoculate the main cultures. Cultures were performed in 500 ml of medium in a 1-l Biostat A Plus bioreactor (Sartorius BBI, Germany) at 37°C, pH 7.2. Dissolved oxygen tension (DOT) was measured using a polarographic sensor (USA). DOT was controlled at 30% air sat. by a varying the stirring rate. Off-gas composition was monitored through a BlueInOne Ferm (BlueSens, Germany) gas analyzer. OTR and CTR were calculated with the proper mass balances [20]. Three independent cultures under each condition were performed.

Active VHb was measured by CO-difference spectral analysis as described by Yang *et al*. [11] from samples taken during the exponential growth phase and lysed with lysozyme. The CO-difference spectra was measured with an UV-Vis Evolution 300 Spectrophotometer (Thermo Scientific) using the extinction coefficient *ε*_(419–436 nm)_ = 274 mM^-1^ cm^-1^ [11]. Biomass concentration was measured as dry cell weight. Glucose was quantified in an YSI 2700 biochemistry analyzer (YSI Inc., USA). Acetate, lactate and succinate were quantified using enzyme kits (R-Biopharm, Germany).

### Transcriptional Analysis

RNA extraction, purification and quality control, as well as cDNA synthesis were performed as previously detailed [20]. RNA integrity numbers of the purified samples were between 7.8 and 9.2. cDNA was synthesized using the primers listed in Supplementary Table 1. The gene *gmk* (coding for guanylate kinase) was selected from 9 candidates as the internal control gene, due to a nearly constant Cq value in all strains (Table S2). RT-qPCR was performed as described before [21], and the transcription levels calculated after Livak and Schmittgen [22]. These experiments were compliant with MIQE guidelines [23].

## Supplemental Materials



Supplementary data for this paper are available on-line only at http://jmb.or.kr.

## Figures and Tables

**Fig. 1:**
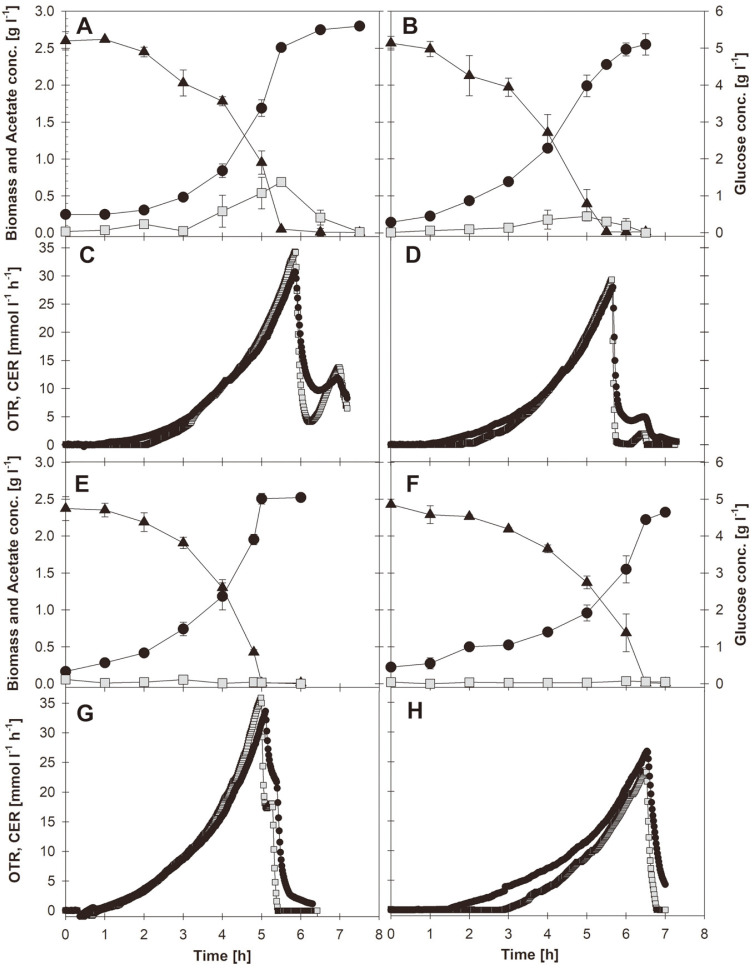
Growth profiles of the engineered *E. coli* strains. A: Cell growth (circles), glucose (triangles) and acetate concentration (squares) in cultures of strain W3110 *recA*^-^; B: Cell growth (circles), glucose (triangles) and acetate concentration (squares) in cultures of strain W3110*recA*^-^ *vgb*^+^; C: Oxygen transfer rate (OTR, squares) and carbon dioxide evolution rate (CER, circles) in cultures of strain W3110 *recA*^-^; C: Oxygen transfer rate (OTR, squares) and carbon dioxide evolution rate (CER, circles) in cultures of strain W3110 *recA*^-^ *vgb*^+^; E: Cell growth (circles), glucose (triangles) and acetate concentration (squares) in cultures of strain BL21 *recA*^-^; F: Cell growth (circles), glucose (triangles) and acetate concentration (squares) in cultures of strain BL21 *recA*^-^ *vgb*^+^; G: Oxygen transfer rate (OTR, squares) and carbon dioxide evolution rate (CER, circles) in cultures of strain BL21 *recA*^-^; H: Oxygen transfer rate (OTR, squares) and carbon dioxide evolution rate (CER, circles) in cultures of strain BL21 *recA*^-^ *vgb*^+^. Cultures were carried out in mineral medium supplemented with 5 g l^-1^ of glucose under aerobic conditions (DOT ≥ 30% air sat.) and pH 7.0 in 1 l bioreactors. Vertical lines represent the experimental deviation between triplicate cultures.

**Fig. 2:**
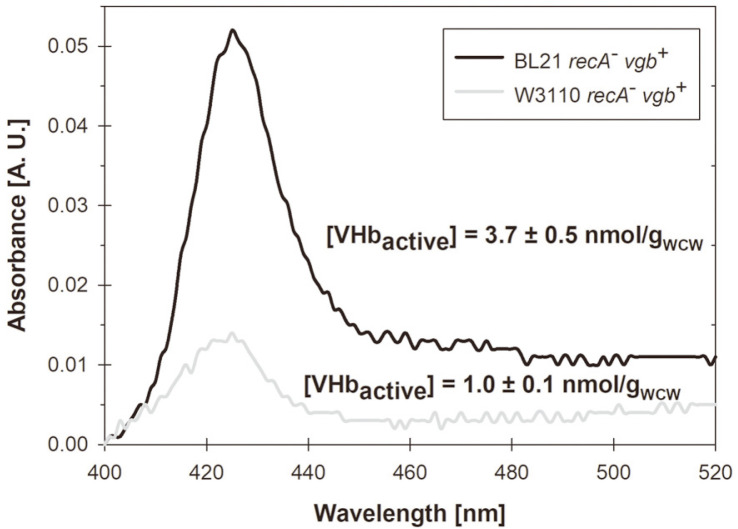
Typical CO differential spectra of *E. coli* strains BL21 (upper line) and W3110 (lower line) constitutively expressing VHb. Samples were taken from aerobic cultures and spectral analyses were performed from the lysate of 0.5 g of wet cells. Average and standard deviation includes the results from triplicate cultures.

**Fig. 3:**
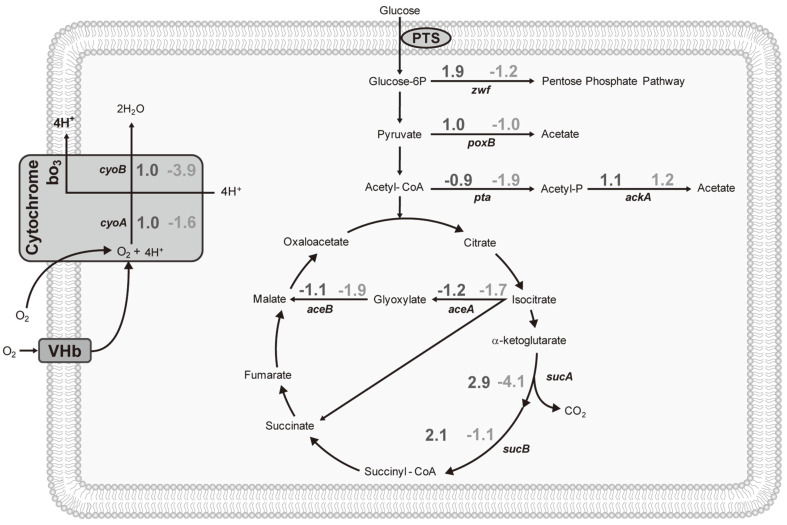
Expression levels of strains W3110*vgb*^+^ relative to W3110*vgb*^-^ (left numbers) and BL21 *vgb*^+^ relative to BL21*vgb*^-^ (right numbers). The results are shown above the arrows of the reactions where the transcription product is involved. Standard deviation ranges between 4 and 20%.

**Table 1:** Kinetic and stoichiometric parameters of the wild type and engineered strains.

Parameter/Strain	W3110	W3110*vgb*^+^	BL21	BL21*vgb*^+^
*µ* (h^-1^)	0.53 ± 0.01	0.53 ± 0.02	0.54 ± 0.00	0.46 ± 0.02
*Y_x/s_* (g g^-1^)	0.49 ± 0.02	0.43 ± 0.03	0.44 ± 0.00	0.41 ± 0.00
*Y_acet/S_* (g g^-1^)	0.13 ± 0.01	0.09 ± 0.01	0.05 ± 0.01	0.02 ± 0.00
*-q_S_* (g g^-1^ h^-1^)	1.10 ± 0.07	1.31 ± 0.08	1.23 ± 0.00	1.13 ± 0.05
*q_acet_* (g g^-1^ h^-1^)	0.16 ± 0.01	0.10 ± 0.01	0.05 ± 0.01	0.02 ± 0.00
*q_lac_* (g g^-1^ h^-1^)	0.008 ± 0.001	0.010 ± 0.001	0.002 ± 0.000	0.049 ± 0.006
*q_suc_* (g g^-1^ h^-1^)	0.012 ± 0.001	0.015 ± 0.001	0.002 ± 0.000	0.001 ± 0.000
*-q_O_2__* (mmol g^-1^ h^-1^)	15.0 ± 0.5	18.3 ± 1.1	19.9 ± 2.1	15.4 ± 0.4
*q_C_*_O_2__ (mmol g^-1^ h^-1^)	13.1 ± 0.7	15.9 ± 1.0	18.3 ± 1.8	15.0 ± 0.8
*q_ATP_* (mmol g^-1^ h^-1^)^a^	44.7 ± 1.49	54.5 ± 3.0	59.3 ± 6.3	45.9 ± 1.19
RQ	0.87 ± 0.03	0.87 ± 0.02	0.92 ± 0.02	0.94 ± 0.00

Data correspond to the mean of duplicate cultures and were calculated during the exponential growth phase. m: specific growth rate; Yx/s: biomass yield from glucose; Yacet/S: acetate yield from glucose; *q_S_*: specific glucose consumption rate; *q_acet_*: specific acetate production rate; qlac: specific lactate production rate; qsuc: specific succinate production rate; *q_O2_*: specific oxygen uptake rate; *q_CO2_*: specific carbon dioxide production rate. *q_ATP_* was calculated with an stoichiometry of 1.49 mol ATP/1/2 molO_2_ [13].
