# Supplementary material for: Physiological Response of Escherichia coli W3110 and BL21 to the Aerobic Expression of Vitreoscilla Hemoglobin
Source: J Microbiol Biotechnol. 2020 Jul 14;30(10):1592–6. doi: 10.4014/jmb.2004.04030 (PMC9728183; doi:10.4014/jmb.2004.04030)
Supplement: Supplementary file 1 [file JMB-30-10-1592-supple.pdf]

J. Microbiol. Biotechnol.
https://doi.org/10.4014/jmb.2004.04030

Supplementary Table 1. Gene name, coding enzyme and primer sequences used for PCR amplification and RT-qPCR assays in *E. coli* BL21*recA*⁻ and W3110*recA*⁻ expressing or non-expressing the VHb.

Gene	Coding enzyme	Forward primer sequence	Reverse primer sequence
ackA	Acetate kinase	CTGGTTCTGAACTGCGGTAGTTC	GGCAGGTGGAAACATTCGG
poxB	Pyruvate dehydrogenase	AAAAGCCGATCGCAAGTTTC	GGTGAATGGCTTTCTCGCTC
pta	Phosphate acetyltransferase	ACAATGTTGATCCGGCGAAG	CATATCGATCGCACGAGTCG
aceA	Isocitrate lyase	ACATGGGCGGCAAAGTTTTA	AACCAGCAGGGTTGGAACG
aceB	Malate sintase A	GAACTGGCTTTCACAAGGCC	TGTGGCGTAAAATGCGTCAC
sucA	2-oxoglutarate dehydrogenase E1 component	GCGGCAAAGAAACCATGAAA	TTCGGTGCTGGTAATGTGCA
sucB	Dihydrolipoyllysine-residue succinyltransferase component of 2-oxoglutarate dehydrogenase complex	GCAGTACGGTGAAGCGTTTG	CTTCCG GGTAACGTTTCAGG
cyoA	Cytochrome bo_3 ubiquinol oxidase subunit 2	GGCATTGCTACCGTGAATGA	AGACGCGGAATGAAGAAGGA
cyoB	Cytochrome bo_3 ubiquinol oxidase subunit 1	CTGACCTCCGTCGACCATAAA	TGGCTACGCATCATAATGGC
zwf	Glucose-6-phosphate 1-dehydrogenase	GCACGCGTAGTCATGGAGAA	CGGTAAACCTGGCACTCCTC

Supplementary Table 2. *Cq* values of the genes analyzed as candidates for internal reference control for BL21 and W3110 strains. *gyrA* DNA girase A, *gyrB* DNA girase B, *secA* protein translocase sub A, *dnaG* DNA primase, *adk* adenilate kinase, *gmk* guanilate kinase, *ftsZ* cellular division protein, *recA* recombinase A, *rpoB* ARN polimerase β-subunit , *rpoC* ARN polimerase ´β-subunit.

Gene	BL21*recA*$^-$		BL21*recA*$^-$*vgb*$^+$		Mean	SD
gyrA	0	0	0	0	0.00	0.00
gyrB	0	0	23.87	0	5.97	11.94
secA	0	0	0	0	0.00	0.00
dnaG	30.27	23.75	21.83	21.14	24.25	4.16
adk	19.77	20.25	18.81	18.62	19.36	0.78
gmk	**20.55**	**21.17**	**20.07**	**19.61**	**20.35**	**0.67**
ftsZ	17.17	18.17	18.72	17.97	18.01	0.64
recA	27.53	26.78	28.12	27.94	27.59	0.60
rpoB	19.84	18.29	19.18	19.32	19.16	0.64
rpoC	17.74	17.81	17.57	17.57	17.67	0.12

Gene	W3110*recA*$^-$		W3110*recA*$^-$*vgb*$^+$		Average	SD
gyrA	0	0	0	0	0.00	0.00
gyrB	26.74	22.6	21.35	20.16	22.71	2.86
secA	0	0	0	29.61	7.40	14.81
dnaG	22.17	22.63	20.68	21.78	21.82	0.83
adk	20.08	18.49	17.86	18.27	18.68	0.97
gmk	**21.03**	**19.94**	**20.27**	**19.68**	**20.23**	**0.59**
ftsZ	19.95	19.25	18.84	18.94	19.25	0.50
recA	28.16	27.95	28.27	27.38	27.94	0.40
rpoB	25.78	18.83	17.79	17.94	20.09	3.82
rpoC	18.41	18.62	18.1	17.58	18.18	0.45
